# Bi-allelic missense variants in human *GPN2* result in Perrault syndrome

**DOI:** 10.1016/j.ajhg.2026.05.016

**Published:** 2026-07-02

**Authors:** Thomas B. Smith, Rabia Faridi, Leigh A.M. Demain, Sayaka Inagaki, Yasuko Ishibashi, Huw B. Thomas, Inna A. Belyantseva, Alessandro Rea, King Lam Lai, Arshia Maqbool, Bushra Rauf, Muhammad Asad Usmani, Alejandro A. Schäffer, Robert J. Morell, Andrew Green, Sondhya Ghedia, Mathilda Wilding, Robin Hay, Yuliya Sokolova, Gavin P. Riordan, Isabelle Schrauwen, Khurram Liaqat, Saima Riazuddin, Zubair M. Ahmed, Wade W. Chien, Langping He, Amanda McGovern, Helen Byers, Glenda M. Beaman, Wasim Ahmad, Suzanne M. Leal, Robert W. Taylor, Sheikh Riazuddin, Raymond T. O’Keefe, Thomas B. Friedman, William G. Newman

**Affiliations:** 1 Division of Evolution, Infection and Genomics, School of Biological Sciences, Faculty of Biology Medicine and Health, University of Manchester, Manchester M13 9PL, UK; 2 Manchester Centre for Genomic Medicine, St Mary’s Hospital, Manchester University NHS Foundation Trust, Manchester M13 9WL, UK; 3 Laboratory of Molecular Genetics, National Institute on Deafness and Other Communication Disorders, National Institutes of Health, Bethesda, MD 20892, USA; 4 Inner Ear Gene Therapy Program, NIDCD, NIH, Bethesda, MD 20892, USA; 5 Jinnah Burn and Reconstructive Surgery Centre, Allama Iqbal Medical Research Center, University of Health Sciences, Lahore 54500, Pakistan; 6 Cancer Data Science Laboratory, National Cancer Institute, Bethesda, MD 20892, USA; 7 Genomics and Computational Biology Core, National Institute on Deafness and Other Communication Disorders, National Institutes of Health, Bethesda, MD 20892, USA; 8 Department of Clinical Genetics, Children’s Health Ireland at Crumlin, D12 N512 Dublin, Ireland; 9 Department of Clinical Genetics, Royal North Shore Hospital, St Leonards, NSW 2065, Australia; 10 Advanced Imaging Core, National Institute on Deafness and Other Communication Disorders, National Institutes of Health, Bethesda, MD 20892, USA; 11 Department of Translational Neurosciences, University of Arizona College of Medicine Phoenix, Phoenix, AZ 85004, USA; 12 Center for Statistical Genetics, Gertrude H. Sergievsky Center, and the Department of Neurology, Columbia University Medical Center, New York, NY 10032, USA; 13 Department of Biochemistry, Faculty of Biological Sciences, Quaid-i-Azam University, Islamabad 45320, Pakistan; 14 Department of Otorhinolaryngology Head and Neck Surgery, University of Maryland School of Medicine, Baltimore, MD 21201, USA; 15 Department of Molecular Biology and Biochemistry, School of Medicine, University of Maryland, Baltimore, MD 21201, USA; 16 NHS Highly Specialised Rare Mitochondrial Disorders Service, Newcastle upon Tyne Hospitals NHS Foundation Trust, Newcastle upon Tyne NE2 4HH, UK; 17 Mitochondrial Research Group, Clinical and Translational Research Institute, Faculty of Medical Sciences, Newcastle University, Newcastle upon Tyne NE2 4HH, UK; 18 Genome Editing Unit Core Facility, Faculty of Biology, Medicine and Health, University of Manchester, Manchester M13 9PT, UK; 19 Taub Institute for Alzheimer’s Disease and the Aging Brain, Columbia University Medical Center, New York, NY 10032, USA

## Main text

Perrault syndrome (MIM: 233400, PRLTS1) is a rare autosomal recessive condition characterized by sensorineural hearing loss (SNHL) in both sexes and primary ovarian insufficiency (POI) in 46,XX karyotype females.^1^ Neurological features, including learning disability, developmental delay, ataxia, and leukodystrophy, also occur in some affected individuals. PRLTS1 is genetically heterogeneous, with bi-allelic variants in 13 genes definitively associated with PRLTS1.^2^ Most PRLTS1-associated genes encode proteins important for mitochondrial protein translation or peroxisomal function^1^; nevertheless, many individuals with PRLTS1 remain without a confirmed genetic diagnosis.^1^^,^^2^ Here, we report three families affected by PRLTS1 whose diagnosis could not be explained by variants in the previously reported PRLTS1 genes or in genes associated with SNHL or POI. Through the application of homozygosity mapping and exome sequencing (ES), we identified homozygous missense variants in *GPN2* (MIM: 621544) in the affected individuals in all three families.

**Figure 1:**
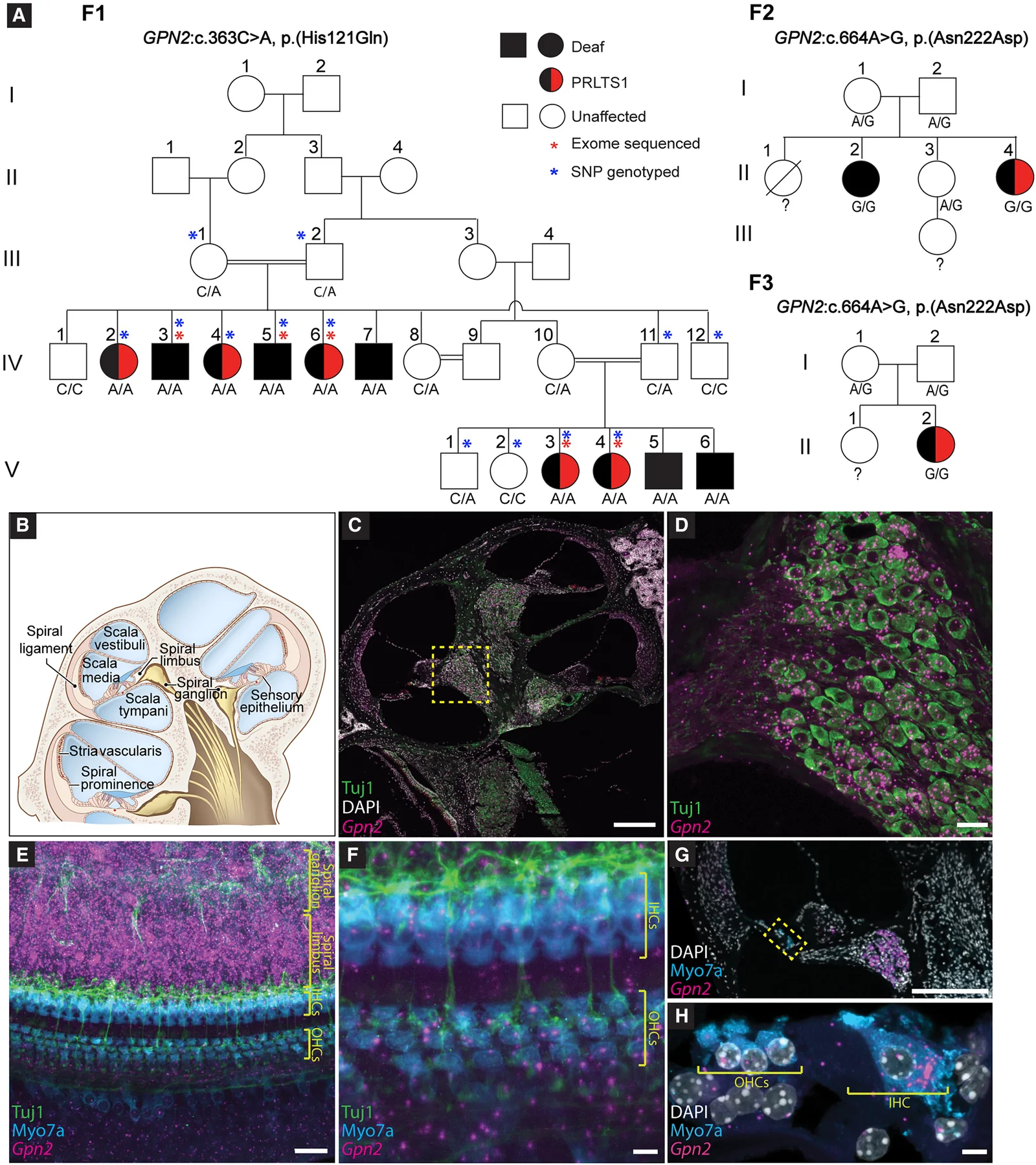
*GPN2* variants in humans that are associated with Perrault syndrome, and *Gpn2* expression in the spiral ganglion neurons, hair cells, and supporting cells of the mouse inner ear (A) Pedigrees of families F1, F2, and F3 showing segregation of variants annotated in accordance with the *GPN2* reference sequence, NCBI RefSeq: NM_018066.4. Clinical details for the affected individuals can be found in the Dryad database. (B) Schematics of cochlea cross-section showing main compartments and anatomical structures. (C) Localization of Gpn2 mRNA in spiral ganglion, lateral wall, and sensory epithelium of the P20 C57BL6 male mouse cochlea cross-section using a *Gpn2* RNAscope probe (magenta). The specimen was co-immunostained with Tuj1 antibody (green) and counter-stained with DAPI (white). (D) Higher-magnification image of the boxed area in (C) (yellow dash line) shows signals of *Gpn2* (magenta) in spiral ganglion neurons highlighted by Tuj1 antibody (green). (E) Flattened z stack of whole-mount sensory epithelium of the cochlea from P20 C57BL6 female mouse shows *Gpn2* (magenta) is concentrated in spiral limbus and spiral ganglion. An antibody against myosin 7a (blue) was used to visualize hair cells. Tuj1 (green) visualized spiral ganglion neurons (applies to [E] and [F]). (F) *Gpn2* expression (magenta) in inner and outer hair cells of a P20 C57BL6 female mouse organ of Corti. (G) Cross-section of the organ of Corti sensory epithelium of P90 C57BL6 female mouse. DAPI (white) and myosin 7a (blue) were used as markers for nuclei and hair cells, correspondingly, together with the *Gpn2* RNAscope probe (magenta). (H) Higher-magnification image of boxed area in (G) showing *Gpn2* signals (magenta) in inner and outer hair cells. Scale bars in (C) and (G) are 200 μm, (D) and (E) are 20 μm, and (F) and (H) are 5 μm.

Family 1 is a consanguineous Pakistani family (PKDF1779) comprised of 10 affected individuals (Figure 1A) presenting with congenital profound SNHL and POI with elevated plasma levels of follicular stimulating hormone (FSH) and luteinizing hormone (LH) with reduced estradiol levels, consistent with hypergonadotropic hypogonadism. Clinical and biochemical details are available on the Dryad database. The Infinium Global Diversity Array-8 v1.0 microarray (Illumina, San Diego, CA), including 1,825,277 markers, was used to perform genome-wide SNP genotyping on genomic DNA samples. A shared homozygous region on chromosome 1 was noted among the seven affected members of Family 1. The multipoint LOD score is 6.03, indicating significant genetic linkage. ES identified a putative causal substitution in *GPN2* (c.363C>A [GenBank: NM_018066.4] [p.His121Gln]) within the linkage interval, which segregated with the phenotype. The p.His121Gln variant is absent in gnomADv4.1.

Family F2 is Irish, comprising two affected sisters with bilateral profound SNHL, POI, and cerebellar ataxia. Family F3, identified through GeneMatcher, is Australian of Irish ancestry, with one affected female proband presenting with profound SNHL, primary amenorrhea, and mild intellectual disability. Intersection analysis of ES data identified a homozygous missense variant, *GPN2* (c.664A>G [GenBank: NM_018066.4] [p.Asn222Asp]), in all three affected individuals, which segregated with the phenotype in both families. Haplotype analysis in the affected members of families F2 and F3 revealed a shared homozygous region of 662 kb flanked by rs9438620 and rs17162431 encompassing the *GPN2* locus, indicative of a shared ancestor. The p.Asn222Asp variant is found at low frequencies in population databases (gnomADv4.1: 0.0003763), but never as homozygous. This variant allele frequency is consistent with a rare autosomal recessive condition. No additional candidate variants in known PRLTS1 genes or genes associated with SNHL or POI were identified in the affected individuals. Conservation of the altered residues in human GPN2 across multiple species, including *Drosophila melanogaster* for both variants, supports their importance for GPN2 function. *In silico* predictors of variant effect provide varied assessments, but Combined Annotation Dependent Depletion scores of 20.7 (His121Gln) and 23.8 (Asn222Asp) are consistent with a deleterious effect.

Human *GPN2* encodes GPN-loop GTPase (GPN)2, comprised of 310 amino acids. The GPN protein family is comprised of GPN1, GPN2, and GPN3, with protein sequence highly conserved in eukaryotes. In *Saccharomyces cerevisiae*, GPN2 is involved in the cytoplasmic assembly of RNA polymerase II (RNAPII) and RNAP III complexes, with no role in subsequent nuclear transport.^3^ It was proposed that GPN2 function is specific for RNAP II and III biogenesis, as almost exclusively RNAP II and III subunit proteins were mislocalized to the cytoplasm of GPN2-mutant yeast cells.^3^ Currently, there is no evidence to suggest that GPN2 has a mitochondrial or peroxisomal function, which is interesting considering that PRLTS1 is often a disorder affecting these organelles and suggests a previously unrecognized mechanism resulting in the PRLTS1 phenotype.

Variants in *GPN1*, *GPN2*, and *GPN3* have not previously been associated with a Mendelian disease. However, variants in many components of RNAPII, including *POLR2A* (MIM: 180660) and *POLR2C* (MIM: 180663), have been associated with leukodystrophy, SNHL, and POI.^4^^,^^5^ Due to the predicted essential functions of RNAP assembly and biogenesis, and lack of bi-allelic loss-of-function *GPN2* variants in population datasets, we postulate that PRLTS1-associated *GPN2* variants are hypomorphic. Deciphering the mechanisms of GPN2-mediated hearing loss in affected individuals, as well as assessment of GPN2 localization and function in ovarian tissues, will be important for development of therapies for PRLTS1. In the mouse inner ear, *Gpn2* mRNA (Figures 1B–1H) was detected in spiral ganglion neurons (SGNs), auditory hair cells, and supporting cells, consistent with the previous finding that multiple other PRLTS1-associated genes are highly expressed in SGNs.^1^

In summary, we present genetic data to support the association of rare or previously unreported bi-allelic missense variants of *GPN2* with predicted deleterious effects in 13 individuals with PRLTS1 from three unrelated families. These data expand the rich genomic architecture of this rare, genetically heterogeneous condition and propose a previously unsuspected mechanism resulting in the PRLTS1 phenotype.

## Acknowledgments

In 2024, Professor Sheikh Riazuddin, PhD, our collaborator and dear colleague, passed away. This study is dedicated to his memory. He was the founding director of the world-class Centre of Excellence in Molecular Biology and Centre for Applied Molecular Biology at the University of the Punjab, Pakistan.

We thank the families for their participation. All study subjects or their legal guardians provided written informed consent in accordance with local regulations. Ethical approval for this study was granted by the National Health Service Ethics Committee (16/WA/0017) and University of Manchester, by the Lahore Pakistan Institutional Review Board (IRB), and the IRB of the National Institutes of Health, USA (OH93-DC-0016). We thank Drs. Dennis Winkler and Mhamed Grati for their critiques of our manuscript.

This study was supported by the Medical Research Council (MR/W019027/1); Royal National Institute for the Deaf (S60_Newman, S35); Action Medical Research (GN2494); NIHR Manchester Biomedical Research Centre (NIHR203308); the Wellcome Trust Centre for Mitochondrial Research (to R.W.T.) (203105/Z/16/Z); the UK NHS Highly Specialised “Rare Mitochondrial Disorders of Adults and Children” Service; The Lily Foundation and LifeArc; and the National Institutes of Health (NIH), National Institute on Deafness and Other Communication Disorders (RO1 DC003594 to S.M.L.), NIDCD/NIH (R01DC012564 to Z.M.A.). This research was supported in part by the Intramural Research Program (DC000039 to T.B.F., unnumbered to A.A.S.) of the NIH. The contributions of the NIH authors are considered Works of the United States Government. This work utilized the computational resources of the NIH HPC Biowulf cluster (https://hpc.nih.gov). We thank the Genomics and Computational Core (ZIC DC000081) and the Advanced Imaging Core (ZIC DC000081) at the NIDCD/NIH. The findings and conclusions presented in this paper are those of the authors and do not necessarily reflect the views of the NIH or the U.S. Department of Health and Human Services.

## Declaration of interests

The authors declare no competing interests.
